# Characteristics of Long COVID: Cases from the First to the Fifth Wave in Greater Tokyo, Japan

**DOI:** 10.3390/jcm11216457

**Published:** 2022-10-31

**Authors:** Kouichi Hirahata, Nobutoshi Nawa, Takeo Fujiwara

**Affiliations:** 1 Hirahata Clinic, Tokyo 150-0002, Japan; 2 Department of Global Health Promotion, Tokyo Medical and Dental University (TMDU), 1-5-45 Yushima, Bunkyo-ku, Tokyo 113-8519, Japan

**Keywords:** long COVID, activities of daily living, performance status

## Abstract

Purpose: Approximately 25–60% of COVID-19 patients develop long-term sequelae of the condition known as long COVID. This study aimed to examine sociodemographic and clinical characteristics of long COVID in Japan. Methods: The data of long COVID patients, defined as those who were symptomatic after 28 days from onset, were collected in an outpatient clinic in Tokyo, Japan between 6 January 2020 and 2 October 2021 (N = 1891). Information on age, sex, employment, infection waves, vaccination, impairment in activities of daily living, and symptoms were obtained from electronic medical records. We used linear regression to analyze the association of patients characteristics with performance status. Results: The mean number of days from onset was 77.6 (SD: 71.3). Female, those who had their work hours reduced, on leave, dismissed or retired or not working, were associated with lower performance status. Fatigue, depressive symptom, brain fog, dyspnea, palpitation, body pain, loss of appetite, fever—but not headache, insomnia, loss of smell, loss of taste, hair loss, or cough—were associated with the lower performance status. Conclusion: Sex and employment status were associated with lower performance status in long COVID patients. Studies are needed to elucidate the full picture of the characteristics of long COVID patients.

## 1. Introduction

COVID-19 has spread worldwide, infecting about 430 million people and killing about six million as of 1 March 2022 [1]. Patients with COVID-19 may have characteristic pulmonary lesions [2], and it is known that some patients with severe acute disease may still have pulmonary lesions 3 months or 1 year after infection [3,4]. Furthermore, several meta-analyses and systematic reviews have reported that about 25–60% of COVID-19 patients develop long-term sequela of the condition, called long COVID [5,6,7,8,9,10]. WHO defines long COVID as “Post COVID-19 condition, also known as long COVID, occurs in individuals with a history of probable or confirmed SARS-CoV-2 infection, usually 3 months from the onset of COVID-19. Symptoms last for at least 2 months and cannot be explained by an alternative diagnosis. Common symptoms include fatigue, shortness of breath and cognitive dysfunction, as well as others that generally have an impact on everyday functioning. Symptoms may appear following initial recovery from an acute COVID-19 episode, or persist from the initial SARS-CoV-2 infection” [11]. As stated in the WHO definition, the most common symptoms include dyspnea, fatigue, hair loss, altered taste, altered smell, and brain fog [5,6,7,8,9]. A small Japanese study of 87 patients reported similar findings, with fatigue being the most common symptom, followed by altered sense of smell, altered sense of taste, and hair loss [12].

The identification of the characteristics of patients with severe long COVID symptoms may provide important information for the identification of high-risk groups and the biological mechanism of long COVID [9,13,14,15,16,17,18,19,20,21,22,23,24,25,26,27,28,29,30]. For example, studies conducted in Saudi Arabia, the United States, England, and Sweden reported positive associations between older age and long COVID [15,17,18,20]. A study from Ghana [13] found that higher educational attainment was protective against long COVID and that there is no association between employment status and long COVID. There have also been studies conducted in various countries such as China, Saudi Arabia, the United States, France, Spain, Germany, England, Sweden, Norway, and Russia which reported that being female is a risk factor for developing long COVID [9,15,18,19,20,22,23,24,26,27,28]. However, two studies from China and Spain reported no association between gender and long COVID [29,30], while the study from Ghana reported a positive association between being male and long COVID [13]. Evidently, the findings on sociodemographic characteristics are still inconclusive and further studies are needed to characterize long COVID.

Japan is known for its low prevalence and low mortality rate of COVID-19 [31], especially up to the fifth wave (before the occurrence of Omicron variant in the sixth wave). Given a positive association between the severity of the acute phase of COVID-19 and the development of long COVID [17,25], the characteristics of long COVID in Japan may be different from those in other countries. However, there have been no large-scale studies on long COVID in Japan. Therefore, the aim of this study was to clarify the characteristics of long COVID in Japan, especially in COVID-19 patients up to the fifth wave before the appearance of the Omicron variant in the sixth wave.

## 2. Methods

### 2.1. Sample

All patients with long COVID, defined as those who were symptomatic after 28 days from onset, were collected in an outpatient clinic in Tokyo, Japan between 6 January 2020 and 2 October 2021. While WHO states that symptoms last at least 2 months [11], the CDC maintains that at least 4 weeks after infection is the beginning of the first identifiable long COVID conditions [32]. Thus, in order to identify long COVID patients from an early stage, the CDC’s 4 weeks was used to define patients in this study.

Among the 1898 patients, we excluded seven whose data on symptoms were not available. The resulting analytical sample included 1891 patients.

### 2.2. Sociodemographic Characteristics

Information on age, sex, employment status, infection waves, date of onset, date of first visit, and vaccination status at the time of the first visit was obtained from electronic medical records. The infection wave classification was based on the date of onset and the past course of infection spread in Japan. Specifically, the onset of infection from 1 January 2020 (start of the study period) to 14 May 2020, from 15 May 2020 to 30 September 2020, from 1 October 2020 to 28 February 2021, from 1 March 2021 to 11 July 2021, and from 12 July 2021 to 2 October 2021 (end of the study period) were classified as the first, second, third, fourth, and fifth infection wave, respectively [33]. The dominant SARS-CoV-2 variant in each infection wave was B.1.1.114 lineage in the first wave, B.1.1.284 lineage and B.1.1.214 lineage in the second wave, B.1.1.214 lineage in the third wave, B.1.1.7 lineage (Alpha variant) in the fourth wave, and B.1.617.2 lineage (Delta variant) in the fifth waves [34,35,36].

### 2.3. Clinical Symptoms

Information on the presence or absence of subjective symptoms (i.e., self-reported symptoms) by patients at the time of first visit was obtained from electronic medical records. Long COVID symptoms included loss of appetite, loss of taste, loss of smell, fatigue, dyspnea, insomnia, headache, hair loss, fever, palpitation, depressive symptom, cough, brain fog and body pain [8,17].

### 2.4. Performance Status Score

We used performance status (PS) score as the primary outcome, which is a 10-point scale, with higher scores indicating greater impairment in activities of daily living [37,38]. Each level is described below [37,38]: 0: “No complaints; able to carry on with normal activities without fatigue”; 1: “Able to carry on with normal activities, but sometimes feels fatigue”; 2: “Able to carry on with normal activities or to perform active work with effort; requires occasional rest”; 3: “Unable to carry on with normal activities or perform active work; requires rest at home without work several days a month,”; 4: “Unable to carry on with normal activities or perform active work; requires rest at home without work several days a week,”; 5: “Unable to carry on with normal activities or perform active work at all, although able to perform light tasks; requires rest at home without work for several days a week”; 6: “Requires rest without work at home for more than half of the week; able to perform light tasks in good health”; 7: “Unable to carry on with normal activities or perform light tasks at all; able to care for self without assistance”; 8: “Remains in bed for more than half a day; able to care for self to some extent, but requires frequent assistance”; and 9: “Unable to care for self; must remain in bed with day-long assistance”.

### 2.5. Statistical Analysis

Linear regression model was used to examine the association between patients’ characteristics (i.e., age, sex, employment status, infection waves, and vaccination status) and the performance status score. We further developed a model using logistic regression to predict the probability of poor performance status, defined as a PS value of 6 or more using patient characteristics (age, sex, employment status and vaccination). Although machine learning methods may perform well for prediction, they are not suited for interpreting the association between exposure and outcome variables, so logistic regression was used in this study.

All analyses were conducted using STATA 17 (StataCorp LP, College Station, TX, USA).

## 3. Results

Table 1 shows the demographic characteristics of the patients in this study (n = 1891). The average age was 37.8 (SD: 12.2) years. Of the 1891 patients, 1129 (59.7%) were female. Four hundred and forty-nine (23.7%), 269 (14.2%), 395 (20.9%), 157 (8.3%) worked regular hours, had their work hours reduced, on leave, dismissed or retired or not currently working, respectively. About half of the patients from the first to the third wave, and the other half were in the fourth and fifth waves. Only 59 (3.1%) had received at least one COVID-19 vaccine at the time of the first visit. The average days from onset to the first visit was 77.6 (SD: 71.3, range 28–641) days. The average performance status score was 3.1 (SD:2.4, range 0–9) and the average number of symptoms was 8.4 (SD: 3.2, range 1–14). The three most frequent symptoms were fatigue (90.3%), depressive symptom (81.2%), and brain fog (76.2%). Loss of smell was observed in about half of the patients, and loss of taste and hair loss were reported in about 45% of the patients.

Table 2 shows the association between patients’ characteristics and PS score. In model 1, age, sex, and employment status were significantly associated with PS score. Regarding age, compared to 20–29 years old, patients under 15 and 15–19 years of age both showed lower PS score, indicating higher performance status ((coefficient: −0.34 (95% CI: −1.19, 0.52) and −0.17 (95% CI: −0.74, 0.40), respectively), but not reaching statistical significance. Female patients showed a higher PS score (i.e., lower performance status) compared to male patients (coefficient: 0.27 (95% confidence interval (CI): 0.08, 0.47)). Compared to those who worked regular hours, those who had their work hours reduced, those on leave, dismissed, or retired, and those who did not work showed a higher PS score (coefficient: 1.59 (95% CI: 1.27, 1.91), 3.64 (95% CI: 3.35, 3.93), 1.67 (95% CI: 1.22, 2.12), respectively). In model 2, we examine the association between each long COVID symptom and PS score, adjusting for patient characteristics. Fatigue (coefficient: 1.11 (95% CI: 0.77, 1.45)), loss of appetite (coefficient: 0.61 (95% CI: 0.41, 0.81)), and body pain (coefficient: 0.48 (95% CI: 0.28, 0.68)) were the three symptoms most associated with the PS score. Conversely, vaccination was not associated with the PS score. Compared to those in the third wave, those in the second wave showed a lower PS score (coefficient: −0.42 (95% CI: −0.80, −0.05)).

We further developed a model using logistic regression to predict the probability of poor performance status, defined as a PS value of 6 or more using patient characteristics. As shown in Supplementary Table S1, in the model where age, sex, employment status and vaccination were entered simultaneously, the area under the curve (AUC) for predicting poor performance status was 0.79 (95% CI: 0.77–0.81).

## 4. Discussion

In this study, we found that female, those who had their work hours reduced, on leave, dismissed or retired or not working was associated with poor health showing as lower performance status.

Previous studies reported that female is a risk factor for developing long COVID [9,15,18,19,20,22,23,24,26,27,28]; however two other studies reported no such association [29,30], while another reported a positive association between male and long COVID [13]. Thus, while most studies to date indicate that being female is a risk factor for long COVID, findings on the relationship between gender and long COVID are still inconclusive. Our study adds to the literature by showing that female is a risk factor for lower performance status in patients with long COVID.

We also found that those who had their work hours reduced, those who on leave, dismissed or retired had a lower performance status. To date, the association between employment status and long COVID have been understudied. The study from Ghana reported no significant association between employment status and long COVID, although the association between unemployed and long COVID was positive in the analysis of the whole sample [13]. While the direction of causality is not known since our current study is cross-sectional, the relationship between employment and long COVID may represent the social impact of long COVID, where people had to reduce their work, leave, or being dismissed because of long COVID, rather than employment status being a determinant of long COVID. We also found that those not working had lower performance status due to long COVID than those who worked regular hours. It is possible that those who were not working may be in poor health and thus were not working. Further research to elucidate the association between not working and lower performance status is warranted.

This study has several limitations. First, while the large sample size of 1891 patients is a strength, this was a single-center study, and the analysis was performed using data from only long COVID patients. There was no data from a control group. Second, this study was a cross-sectional study, and although we described the characteristics of long COVID patients, we were unable to determine the direction of causality between characteristics and performance status. Third, symptoms were self-reported by patients. Nonetheless, this study examined performance status using a validated scale [37,38] in patients with long COVID. Finally, the vaccination coverage of the study sample was low (3.1%) because the study period was from January 2020 to October 2021 and the COVID-19 vaccination was launched in Japan only from February 2021 with medical personnel being the initial target. It is possible that the characteristics of long COVID among vaccinated individuals may differ.

Nonetheless, our study has several implications. We found that female, those who had their work hours reduced, those who on leave, dismissed or retired or those who not working had lower performance status. Because individuals with these characteristics are more likely to have a long COVID burden on top of the acute symptoms of COVID-19, our findings provide important information for the recommendation of vaccine to individuals with such characteristics. Moreover, our findings suggest these patients should be followed with the caution of long COVID in mind.

## 5. Conclusions

In conclusion, we identified the characteristics associated with lower performance status in patients with long COVID. Our result suggests that female is a risk factor for lower performance status in patients with long COVID. In addition, the relationship between employment and long COVID may represent the social impact of long COVID, where people had to reduce their work, leave, or being dismissed because of long COVID. Further studies are needed to elucidate the full picture of the characteristics of long COVID patients.

## Figures and Tables

**Table 1:** Demographic characteristics of the patients who participated in this study (*n* = 1891).

Variable		N (%) or Mean (SD)
Age (years)	<15	27 (1.4)
	15–19	77 (4.1)
	20–29	440 (23.3)
	30–39	508 (26.9)
	40–49	485 (25.7)
	50–59	291 (15.4)
	≥60	63 (3.3)
	mean (SD)	37.8 (12.2)
Sex	Male	762 (40.3)
	Female	1129 (59.7)
Employment	Working regular hours	449 (23.7)
	Having their work hours reduced	269 (14.2)
	On leave, dismissed or retired	395 (20.9)
	Not working	157 (8.3)
	Unknown/missing	621 (32.8)
Infection waves	1 January 2020–14 May 2020 (corresponding to the first wave)	34 (1.8)
	15 May 2020–30 September 2020 (corresponding to the second wave)	112 (5.9)
	1 October 2020–28 February 2021 (corresponding to the third wave)	791 (41.8)
	1 March 2021–11 July 2021 (corresponding to the fourth wave)	345 (18.2)
	12 July 2021–2 October 2021 (until the end of the study period. This corresponds to the fifth wave)	609 (32.2)
Vaccination	Not vaccinated	1832 (96.9)
	Vaccinated	59 (3.1)
Days from onset	Mean (SD) [range]	77.6 (71.3) [28–641]
Performance status (PS) score ^a^	Mean (SD) [range]	3.1 (2.4) [0–9]
PS	<6	1437 (76.0)
	6+	454 (24.0)
Number of long COVID symptoms	Mean (SD) [range]	8.4 (3.2) [1–14]
Fatigue	Yes	1707 (90.3)
Depressive symptom	Yes	1535 (81.2)
Brain fog	Yes	1441 (76.2)
Headache	Yes	1346 (71.2)
Dyspnea	Yes	1302 (68.9)
Insomnia	Yes	1207 (63.8)
Palpitation	Yes	1166 (61.7)
Body pain	Yes	1146 (60.6)
Loss of smell	Yes	991 (52.4)
Loss of appetite	Yes	957 (50.6)
Loss of taste	Yes	854 (45.2)
Hair loss	Yes	847 (44.8)
Fever	Yes	687 (36.3)
Cough	Yes	628 (33.2)

^a^ Performance Status (PS) score is a 10-point scale, with higher scores indicating greater impairment in activities of daily living [37,38]. Each level is described in Methods. SD, standard deviation.

**Table 2:** Association between patients’ characteristics and performance status score (*n* = 1891).

			Crude		Model 1 ^a^		Model 2 ^b^	
	Mean	SD	β	95%CI	β	95%CI	β	95%CI
Age (years)								
<15	2.6	2.6	−0.47	−1.41, 0.48	−0.34	−1.19, 0.52	0.23	−1.37, 0.14
15–19	2.9	2.6	−0.24	−0.83, 0.35	−0.17	−0.74, 0.40	0.44	−0.87, 0.09
20–29 (Ref)	3.1	2.4	Ref		Ref		Ref	
30–39	3.0	2.4	−0.12	−0.43, 0.20	0.13	−0.14, 0.40	0.06	−0.16, 0.34
40–49	3.1	2.4	−0.03	−0.35, 0.28	0.18	−0.10, 0.45	0.003	−0.22, 0.28
50–59	3.3	2.5	0.19	−0.17, 0.55	0.11	−0.20, 0.42	0.004	−0.26, 0.31
≥60	3.6	2.7	0.52	−0.12, 1.17	0.48	−0.07, 1.04	0.50	−0.10, 0.91
*p* for trend			**0.041**		0.058		0.907	
Sex								
Male (Ref)	2.8	2.4	Ref		Ref		Ref	
Female	3.3	2.5	**0.50**	**0.27, 0.72**	**0.27**	**0.08, 0.47**	0.08	−0.10, 0.26
Employment status								
Working regular hours (Ref)	1.3	1.2	Ref		Ref		Ref	
Having their work hours reduced	2.9	1.8	**1.61**	**1.29, 1.93**	**1.59**	**1.27, 1.91**	**0.97**	**0.68, 1.26**
On leave, dismissed or retired	5.0	2.3	**3.69**	**3.41, 3.98**	**3.64**	**3.35, 3.93**	**2.91**	**2.65, 3.18**
Not working	2.9	2.5	**1.59**	**1.20, 1.97**	**1.67**	**1.22, 2.12**	**1.27**	**0.87, 1.68**
Infection waves								
1 January 2020–14 May 2020 (the first wave)	3.6	2.5	0.57	−0.27, 1.40	0.58	−0.14, 1.30	0.34	−0.31, 0.99
15 May 2020–30 September 2020 (the second wave)	2.3	1.9	**−0.68**	**−1.16, −0.20**	−0.21	−0.62, 0.21	**−0.42**	**−0.80, −0.05**
1 October 2020–28 February 2021 (the third wave) (Ref)	3.0	2.4	Ref		Ref		Ref	
1 March 2021–11 July 2021 (the fourth wave)	3.2	2.4	0.21	−0.10, 0.51	0.02	−0.25, 0.29	−0.11	−0.36, 0.15
12 July 2021–2 October 2021 (the fifth wave)	3.3	2.6	**0.27**	**0.009, 0.52**	0.13	−0.10, 0.36	0.10	−0.13, 0.32
Vaccination								
Not vaccinated (Ref)	3.1	2.4	Ref		Ref		Ref	
Vaccinated	3.2	2.5	0.16	−0.48, 0.79	0.14	−0.41, 0.69	−0.15	−0.64, 0.34
Fatigue	3.4	2.3	**2.84**	**2.49, 3.19**			**1.11**	**0.77, 1.45**
No fatigue (Ref)	0.5	1.6	Ref				Ref	
Depressive symptom	3.4	2.4	**1.90**	**1.63, 2.17**			**0.47**	**0.21, 0.73**
No depressive symptom (Ref)	1.5	2.0	Ref				Ref	
Brain fog	3.5	2.4	**1.67**	**1.42, 1.92**			**0.39**	**0.14, 0.63**
No brain fog (Ref)	1.8	2.2	Ref				Ref	
Headache	3.5	2.4	**1.30**	**1.07, 1.54**			0.15	−0.06, 0.36
No headache (Ref)	2.2	2.3	Ref				Ref	
Dyspnea	3.5	2.4	**1.41**	**1.18, 1.64**			**0.24**	**0.02, 0.46**
No dyspnea (Ref)	2.1	2.3	Ref				Ref	
Insomnia	3.6	2.4	**1.34**	**1.12, 1.56**			−0.02	−0.23, 0.20
No insomnia (Ref)	2.2	2.3	Ref				Ref	
Palpitation	3.6	2.4	**1.44**	**1.22, 1.65**			**0.34**	**0.12, 0.55**
No palpitation (Ref)	2.2	2.3	Ref				Ref	
Body pain	3.7	2.4	**1.45**	**1.23, 1.66**			**0.48**	**0.28, 0.68**
No body pain (Ref)	2.2	2.3	Ref				Ref	
Loss of smell	3.1	2.5	0.10	−0.12, 0.32			0.06	−0.17, 0.30
No loss of smell (Ref)	3.0	2.4	Ref				Ref	
Loss of appetite	3.9	2.4	**1.64**	**1.43, 1.85**			**0.61**	**0.41, 0.81**
No loss of appetite (Ref)	2.3	2.2	Ref				Ref	
Loss of taste	3.2	2.5	**0.29**	**0.07, 0.51**			−0.04	−0.28, 0.19
No loss of taste (Ref)	3.0	2.4	Ref				Ref	
Hair loss	3.4	2.5	**0.56**	**0.34, 0.78**			−0.13	−0.31, 0.06
No hair loss (Ref)	2.8	2.4	Ref				Ref	
Fever	3.9	2.4	**1.20**	**0.98, 1.43**			**0.43**	**0.22, 0.64**
No fever (Ref)	2.6	2.4	Ref				Ref	
Cough	3.5	2.4	**0.60**	**0.37, 0.84**			−0.16	−0.37, 0.05
No cough (Ref)	2.9	2.4	Ref				Ref	

^a^ Model 1 was adjusted for age, sex, employment status, infection waves and vaccination using linear regression model. ^b^ Model 2 adjusted for age, sex, employment status, infection waves and vaccination, fatigue, depressive symptom, brain fog, headache, dyspnea, insomnia, palpitation, body pain, loss of smell, loss of appetite, loss of taste, hair loss, fever, and cough using linear regression model. Bold indicates *p* < 0.05. SD, standard deviation; CI, confidence interval.

## Data Availability

The data is available from the corresponding author upon reasonable request.

## Supplementary Materials

The following supporting information can be downloaded at: https://www.mdpi.com/article/10.3390/jcm11216457/s1, Table S1: Association between patients’ characteristics and poor performance status defined as a performance status score of 6 or more (*n* = 1891).
