# Supplementary material for: Characteristics of Long COVID: Cases from the First to the Fifth Wave in Greater Tokyo, Japan
Source: J Clin Med. 2022 Oct 31;11(21):6457. doi: 10.3390/jcm11216457 (PMC9654540; doi:10.3390/jcm11216457)
Supplement: Supplementary file 1 [file jcm-11-06457-s001.zip › jcm-1996679-supplementary.pdf]

Supplementary Materials

Table S1. Association between patients' characteristics and poor performance status defined as a performance status score of 6 or more (n=1,891).

			Crude		Model 1[a]	
	N	%	OR	95%CI	OR	95%CI
Age (years)						
<15	7	25.9	1.15	0.47, 2.78	1.29	0.50, 3.29
15-19	17	22.1	0.93	0.52, 1.66	0.97	0.50, 1.87
20-29 (Ref)	103	23.4	Ref		Ref	
30-39	113	22.2	0.94	0.69, 1.27	1.18	0.84, 1.66
40-49	112	23.1	0.98	0.72, 1.33	1.19	0.85, 1.68
50-59	80	27.5	1.24	0.88, 1.74	1.22	0.83, 1.77
≥60	22	34.9	1.76	1.00, 3.08	1.96	1.04, 3.68
P for trend			0.085		0.096	
Sex						
Male (Ref)	152	20.0	Ref		Ref	
Female	302	26.8	**1.47**	**1.17, 1.83**	1.28	0.999, 1.64
Employment status						
Working regular hours (Ref)	8	1.8	Ref		Ref	
Having their work hours reduced	29	10.8	**6.66**	**3.00, 14.80**	**6.53**	**2.94, 14.53**
On leave, dismissed or retired	216	54.7	**66.52**	**32.16, 137.58**	**65.28**	**31.52, 135.23**
Not working	37	23.6	**17.00**	**7.71, 37.47**	**17.00**	**7.42, 38.93**
Vaccination						
Not vaccinated (Ref)	439	24.0	Ref		Ref	
Vaccinated	15	25.4	1.08	0.60, 1.96	1.08	0.56, 2.09

[a] In model 1, age, sex, employment status and vaccination were put into model simultaneously.
Bold indicates p<0.05. OR, odds ratio; CI, confidence interval.
